# Solitary fibrous tumor of all sites: outcome of late recurrences in 14 patients

**DOI:** 10.1186/2045-3329-3-4

**Published:** 2013-04-03

**Authors:** Giacomo G Baldi, Silvia Stacchiotti, Valentina Mauro, Angelo P Dei Tos, Alessandro Gronchi, Ugo Pastorino, Leonardo Duranti, Salvatore Provenzano, Andrea Marrari, Michela Libertini, Silvana Pilotti, Paolo G Casali

**Affiliations:** 1 Department of Cancer Medicine, Adult Sarcoma Medical Oncology Unit, Fondazione IRCCS Istituto Nazionale dei Tumori, Via Venezian 1, Milan, 20133, Italy; 2 Department of Pathology, Experimental Molecular Pathology Unit, Fondazione IRCCS Istituto Nazionale dei Tumori, Via Venezian 1, Milan, 20133, Italy; 3 Department of Anatomic Pathology, General Hospital of Treviso, P.zza Ospedale 1, Treviso, 31100, Italy; 4 Department of Surgery, Fondazione IRCCS Istituto Nazionale dei Tumori, Via Venezian 1, Milan, 20133, Italy

**Keywords:** Sarcoma, Solitary fibrous tumor, Hemangiopericytoma, Outcome, Late recurrence

## Abstract

**Background:**

We explore the pattern of late recurrence (LR) in solitary fibrous tumor (SFT), focusing on histopathologic characteristics, clinical presentation and patients (pts) outcome.

**Methods:**

Clinical records of all pts with confirmed pathologic diagnosis of SFT treated at our Institution from 2005 to 2011 were reviewed. We analysed the data of pts who relapsed ≥10 years after initial diagnosis.

**Results:**

A total of 14 pts were identified. The primary site of origin was pleura (5 pts), pelvis (4 pts), head and neck (3 pts) and retroperitoneum (2 pts). Primary tumor was a typical SFT in 5 and a malignant SFT in 7 out of 12 pts whose tumor tissue was available for revision. The median time to first recurrence was 12 years (range 10–23). The first relapse was local in 11 cases, distant in 3. Five pts later developed distant metastases. Four out of 5 cases of typical SFT developed distant metastases in spite of their initial benign aspect. No patient was disease-free at the time of the analyses.

**Conclusion:**

Our series suggests that LR can occur in SFT and some cases can behave aggressively even in the absence of any primary morphologic evidence of malignancy. A prolonged follow-up may be advisable.

## Introduction

Solitary fibrous tumor (SFT) is a rare soft tissue neoplasm, with an incidence of about 0,2/100.000/years. It was called “haemangiopericytoma” by Stout and Murray in 1942. However, the term “SFT” had been introduced by Klemperer and Rabin in 1931, being regarded as a kind of pleural mesothelioma [1]. A consensus on the overlap between haemangiopericytoma and SFT was then developed in the ‘90s [2] (though the 2002 WHO “blue books” still retain the two labels separately at least for some anatomical locations) [3,4]. In practice, haemangiopericytoma and SFT are the same entity, whatever their site of origin, and the former term is currently abandoned (the haemangiopericytoma-like histological pattern being a non-specific feature shared by many neoplasms) [5].

In this regard, in the last WHO classification of bone and soft tissue sarcoma SFT will be a separate nosological entity, the term haemangiopericytoma will be deleted and SFT will be classified as “typical” or “malignant” based on number of mitosis, cellular atypia, presence of necrosis and hypercellularity [6].

The anatomical origin of SFT is almost ubiquitous, as for soft tissue sarcomas in general. Indeed, SFT was originally described in the pleura and then thought to originate from serosal surfaces [5,7]. Finally, it was reported in a set of other anatomical locations, going from the meninges to soft tissues [8,9].

SFTs are classified in “typical” and “malignant” based on the mitotic count (< and ≥4/10 high-power microscopic fields, respectively), the presence of necrosis and nuclear polymorphism. However, a strong correlation between morphology and clinical course is lacking, so that, as of today, there is no way to predict the outcome of a SFT based on its pathologic features.

Indeed, we need such prognosticators, since SFT runs a malignant course at least in 15-30% of cases [8,9]. Furthermore, SFTs can rarely show an abrupt transition from conventional SFTs to high-grade sarcoma, also called “dedifferentiated” SFTs. These “dedifferentiated” lesions are aggressive soft tissue sarcomas [10].

Relatively few published series about SFT [11-19] are available. In general, they prove complete surgical resection, whenever possible, associated with a favourable long-term survival rate as opposed to incomplete excision.

Late recurrences are one of the clinical characteristics of SFT’s [20-27]. Therefore, we decided to search our institutional database and we found 14 SFT patients who relapsed after ≥10 years from first complete surgery and received medical treatment for the disease. This paper reports on these patients.

## Patients and methods

Clinical records of all patients with the diagnosis of SFT who were treated at the Cancer Medical Department of our institution from 2005 to 2011 were reviewed retrospectively. We looked for patients who had relapsed after ≥10 years from initial diagnosis, irrespective of where primary tumor surgery was performed.

In addition, in order to estimate the frequency of late relapses in this histological sarcoma subtype, we also searched the institutional surgical database for all cases of SFT surgically treated from 1995 to 2002. The number of late relapses is provided in this series, while the analysis of the clinical presentation and outcome of relapses is confined to patients treated at our institution.

We selected only patients with initial complete surgery. We reviewed all initial surgical reports, applying current criteria for quality of surgery. Excisions were classified according to the closest surgical margin, defining as R1 those with microscopically infiltrated margins and R0 those with microscopically negative margins.

In 12 cases, the first pathologic diagnosis was subsequently confirmed by experienced pathologists specialized in sarcomas. In 2 cases (case 3 and 14), primary tumor tissue was unavailable for review due to the long time interval. In all cases, we had a pathologic diagnosis of relapse (either following surgical excision or biopsy). Review of the initial tumor was made on tumor tissue samples from primary surgery in all cases. With regard to relapses, tissue was available for all patients but 7, for whom fine needle aspiration cytology (FNAC) was done (case 2, 3, 9, 10 and 14 for first relapses, and case 5, 7 and 14 for second relapses: see Table 1). In 9 cases, unstained sections were available in addition to hematoxilin/eosin (HE), so that the morphologic diagnosis was complemented with CD34/bcl2 in 3 cases, CD34/Ki-67 in 3, CD34 in 2. In all 14 cases operated on at our institution (9 at first surgery and 5 at relapse), we assessed CD34 (clone NCL-L-END, Novocastra; 1:200), bcl (Clone 124; DakoCytomation; 1:500), CD99 (clone MIC2-12E7; Dako; 1:200) and Ki-67 (clone MIB-1; Dakocytomation; 1:200), using Ultra Vision Quanto detection System HPR (Termo Scientific) according to manufacturer’s protocol and antigen retrieval (6^′^ at 95°C 5 mM citrate buffer ph 6 for the first three Abs and 15^′^ at 95°5 mM citrate buffer ph6 for Ki-67). All cases were re-classified according to the updated criteria for the diagnosis of SFT, used at the time of the analyses [4,6,10,28].

**Table 1:** Patient characteristics and pathological/clinical findings

**Patient**	**Gender**	**Age at diagnosis**	**Primary site**	**Histology**	**Type of surgery (R0/R1/R2)**	**Years first relapse**	**Pattern of relapse**	**First relapse surgery**	**Histology**	**Years second relapse**	**Pattern of relapse**	**Second relapse surgery**	**Histology**	**Status last FU**
1	M	52	pelvis	MSFT	R1	10y	local	Y	MSFT	2y	local	Y	MSFT	DOD
2	M	28	head and neck	MSFT	R1	12y	mts	N	MSFT^*^	1y	mts	N	NA	DOD
3	M	48	pleura	NA	R1	11y	mts	N	MSFT*	1y	mts	N	NA	LFU
4	M	31	pelvis	MSFT	R1	10y	local	Y	MSFT	0y	local	Y	MSFT	AWD
5	M	61	pelvis	MSFT	R1	11y	local	Y	MSFT	2y	mts	N	MSFT*	DOD
6	F	36	pleura	SFT	R1	10y	local	Y	MSFT	3y	local	Y	MSFT	LFU
7	F	66	pelvis	MSFT	R1	11y	local	Y	MSFT	1y	mts	N	MSFT*	LFU
8	F	27	head and neck	MSFT	R1	16y	local	Y	MSFT	11y	local	Y	MSFT	AWD
9	F	46	retroperitoneum	SFT	R1	13y	mts	N	PDSFT*	1y	mts	N	NA	DOD
10	F	53	pleura	SFT	R1	12y	local + mts	N	MSFT*	1y	local	N	NA	AWD
11	M	41	pleura	MSFT	R1	20y	local	Y	MSFT	1y	local	N	NA	LFU
12	M	43	pleura	SFT	R1	13y	local	Y	MSFT	1y	local	N	NA	DOD
13	M	58	head and neck	SFT	R1	10y	local	Y	MSFT	6y	mts	Y	MSFT	DOD
14	M	41	retroperitoneum	NA	R1	23y	local	N	MSFT*	1y	mts	N	MSFT*	DOD

*Histological diagnosis was performed by FNAC.

SFT, typical solitary fibrous tumor; MSFT, malignant solitary fibrous tumor; PDSFT, pleomorphic/dedifferentiated solitary fibrous tumor.

NA, not assessed; DOD, dead of disease; LFU, lost to follow-up; AWD, alive with disease.

Type and timing of follow up examinations varied among different Institutions. In the majority of patients, however, total body CT-scan were repeated every six months until the fifth year after the primary excision. Then, X-ray and/or abdominal ultrasound (on the basis of the site of the primary tumor) was repeated every year and CT scan was repeated to confirm recurrence.

Time to first recurrence was defined as the interval between the excision of the primary tumor and the time of first relapse (local and/or metastatic), detected and/or confirmed by CT-scan. We estimated median OS from the date of diagnosis and from the date of first relapse until death from any cause, using the Kaplan-Meier method [29].

This retrospective case series analysis was approved by the Institutional Ethics Committee.

## Results

Patient characteristics are displayed in Table 1.

A total of 9 SFT patients were identified in the institutional surgical database as having had a relapse at an interval of ≥10 years from primary treatment. Only 3 of them are included in this analysis. The number of patients in this database is 83, so the rate of late relapse in our series is 10%.

A total of 14 patients relapsing ≥10 years after complete surgical resection of the primary tumor were identified amongst all soft tissue sarcoma patients treated at our institution.

The primary site of origin was pleura in 5 patients, pelvis in 4, head and neck in 3 (orbital region, ethmoid, maxillary region), retroperitoneum in 2. No patient had a meningeal presentation, probably due to the referral pattern of our institution.

All patients had extra-compartimental disease. Three patients received adjuvant radiotherapy (RT), with a dose ranging from 45 to 60 Gy. No patient was treated with adjuvant chemotherapy.

The median time to first recurrence was 12 years (range: 10–23 years). Ten patients recurred locally without metastatic disease, 1 patient experienced both local and distant relapse, 3 patients had a distant recurrence without local relapse.

The 3 patients with distant recurrence only had previously received adjuvant RT, as opposed to no patient among those with local relapses.

Among 11 patients who recurred locally (70% of the cases in the current series), 9 were re-treated with surgery, consisting in a macroscopic complete excision in all cases. Postoperative RT was added in 3 cases.

Systemic chemotherapy was administered in 6 patients (3 with metastatic disease and 3 with local recurrence), using a regimen with anthracycline and ifosfamide in 3 cases, high-dose continuous infusion ifosfamide in 2 cases, and cisplatin plus gemcitabine in 1 case. The best response by RECIST was a partial response in 1 case (treated with anthracycline plus ifosfamide), stable disease in 3 cases (treated with anthracycline plus ifosfamide and high dose ifosfamide in 2 cases) and progression in 2 cases (treated with anthracycline plus ifosfamide and cisplatin plus gemcitabine respectively).

All patients initially treated with surgery on the first local recurrence subsequently relapsed, either locally or distantly in 6 and 3 cases, respectively.

**Figure 1:**
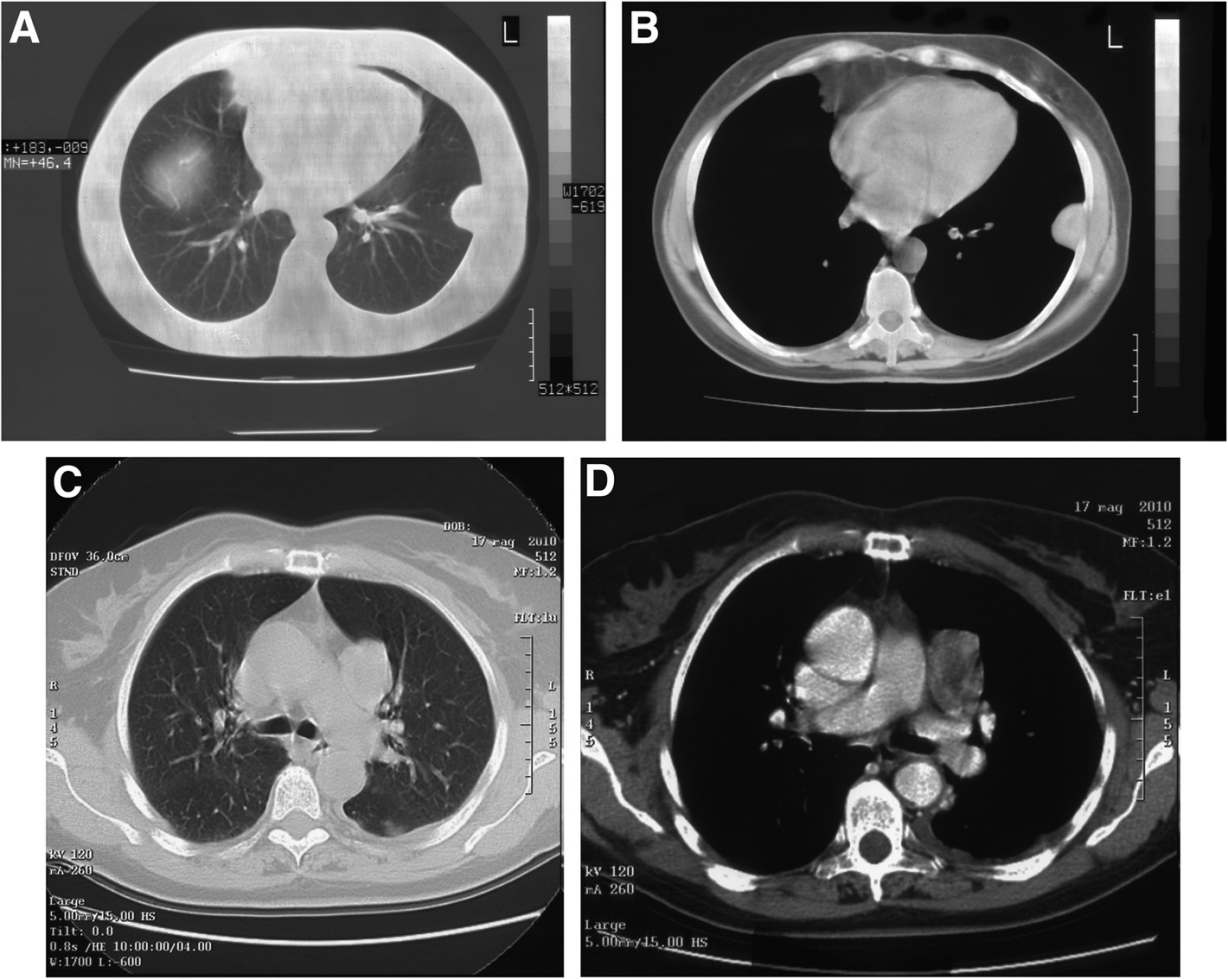
CT scans of primary solitary fibrous tumor of the pleura (1A-B) and the loco-regional relapse in mediastinal pleural (1C-D) after 12 years.

Overall, the median number of relapses per patient all over their clinical history was 2.8 (range: 2–4). Six out of the 14 patients had more than one multifocal loco-regional recurrence (Figure 1A-B-C-D), while 8 developed distant metastases during the course of the disease.

**Figure 2:**
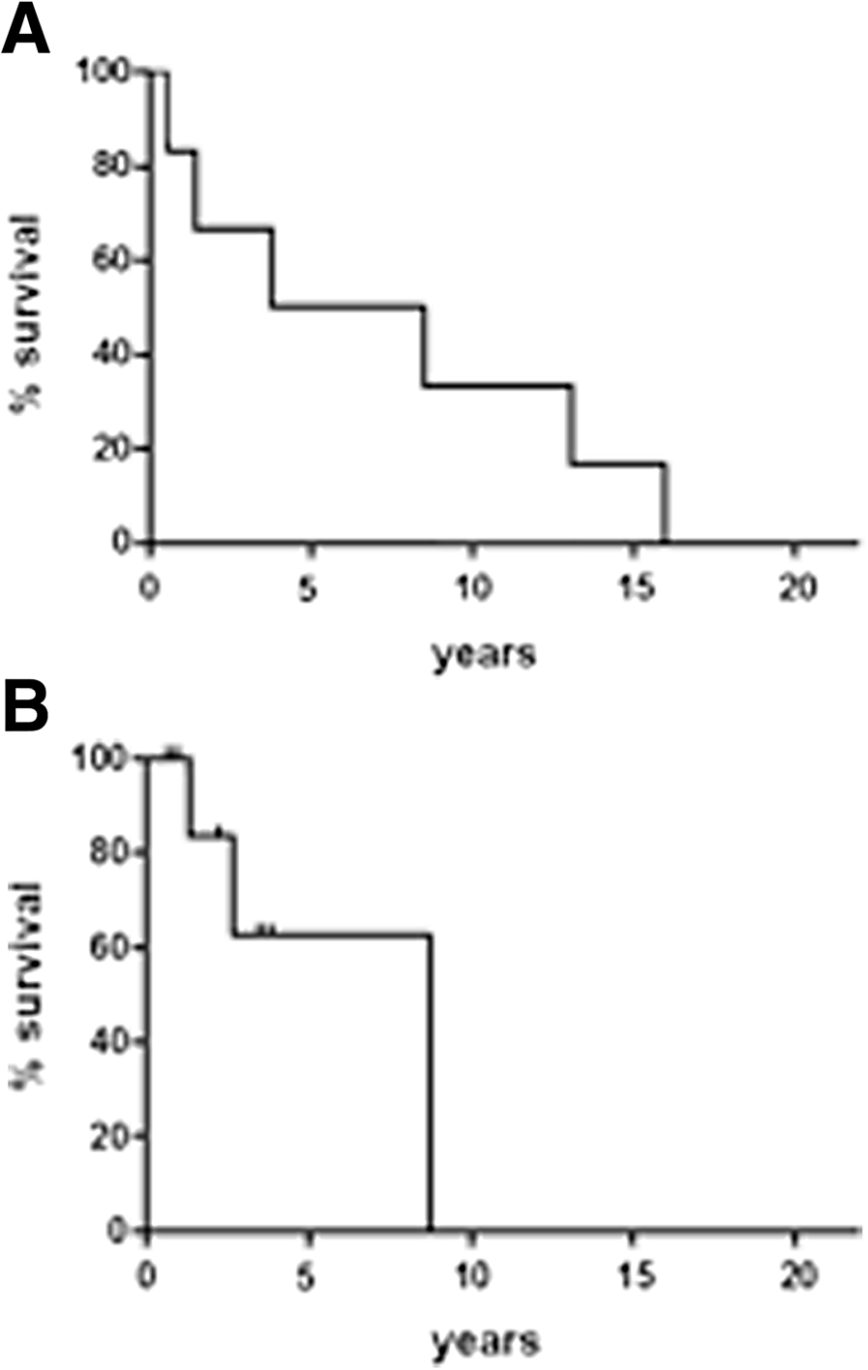
OS curves for patients with local recurrence only (2A) and for those with metastatic ± local recurrence (2B).

Median OS from the first diagnosis was 19 years. Figure 2 displays the OS curves from relapse, for patients with local recurrence only (Figure 2A) and for those with metastatic ± local recurrence (Figure 2B).

At the time of the last follow-up, all patients had evidence of disease: 7 had died of disease; 3 were alive after 2, 5 and 14 years from the first relapse, respectively; 4 patients were lost to follow-up.

On review of the 12 available primary tumor specimens, SFT could be diagnosed as “typical” in 5 cases and “malignant” (MSFT) in 7. Typical cases were collagen-rich and hypocellular, had a very low mitotic rate, and lacked any evidence of cytological atypia or features recalling lipomatous or giant cell variants of SFT [5]. Four cases of typical SFT at first diagnosis had evidence of metastases at their first or second relapse.

**Figure 3:**
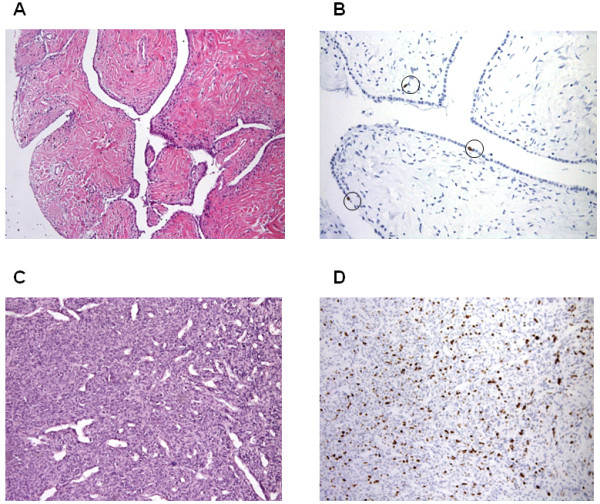
Histopathological pattern of primary typical solitary fibrous tumor (3A) with low expression of Ki-67 (3B) and the malignant counterpart at relapse (3C) with high expression of Ki-67 (3D).

All cases with a typical STF at the time of the primary tumor showed aspects consistent with a MSFT at the time of first and second relapse (Figure 3A-B-C-D), and one patient (#9) whose liver metastasis was assessed by FNAC had a dedifferentiated variant of SFT. This tumor had a growth pattern closely resembling an Ewing sarcoma/pPNET and recapitulating the recently described round-cell dedifferentiated variant of SFT [10]. The correct diagnosis was rendered after ruling out a diagnosis of pPNET by FISH analysis and reviewing the primary tumor, as we already reported [30].

## Discussion

We report on a series of 14 SFT patients who relapsed after ≥10 years from initial diagnosis (median: 12 years) and were treated thereof at our institution. Their median OS from first diagnosis was 19 years and median OS from first relapse was 8 years. Most patients (70%) relapsed at the site of the primary tumor, with only 3 patients recurring at distant sites without evidence of local relapse. Six patients developed metastatic disease only as late recurrence. No patient was cured, even amongst those undergoing complete salvage surgery of their first local recurrence. However, median OS was 8 years from relapse for all patients, with no major difference between locally relapsing and metastatic patients.

SFT represents a very rare disease and few studies are available on the natural history of this tumor. In particular, few cases of late relapses have been reported [20-26]. They were mostly meningeal and pleural SFTs. Interestingly, none of our patients had a meningeal origin, due to the referral pattern of our institution, and some had other than pleural SFTs. Thus, we can conclude that late relapses are a feature of SFT as such, i.e. they are not confined to meningeal or pleural primary sites. This conclusion can be made even on a limited series of patients, picked up at a single institution on the basis of their unfavourable outcome: thus, selection biases should be taken into account when looking at this retrospective case series analysis.

It is well known that currently available pathologic criteria for defining SFT “malignancy” are not satisfactory. Our series confirms that SFTs can have an aggressive behaviour even in the absence of any morphologic evidence of malignancy at onset. Interestingly, all our patients showed signs of malignancy on relapse. This points to a pathologic evolution which takes place in relapsing SFTs, even when the relapse occurs late. In this sense, it is clear that we need to refine criteria for SFT classification, although available pathologic markers of malignancy clearly correspond to a malignant attitude, having being recorded in all our relapses.

However, aside from those rare cases in which a frank sarcomatous evolution is seen, the malignant features of SFT are consistent with a low-aggressiveness tumor. The clinical counterpart of this conclusion is the long OS of relapsing patients in our series, although the long previous disease-free interval is obviously a bias selecting more indolent cases.

The patterns of relapse we observed emphasize the inherent limitations of surgery in SFT, at least of some typical anatomical sites. In fact, most of our patients first recurred with a multinodular loco-regional relapse, and metastases appeared later on. As observed by many [31], high rates of local failure are found in epidural, pleural and pelvic/retroperitoneal SFT, while local failure are much rarer with SFT of soft tissues. In other words, SFT arising in the pleural space, or retroperitoneum, or meninges, may well tend to recur “locally” even when they have a benign aspect and are apparently resected in a complete manner, simply because of the inherent limitations of surgery in such anatomical areas. Furthermore, “local” relapse in the pleural space will inevitably lead to pleural dissemination, thus to a pattern of spread which is very similar to a “metastatic” extent. This explains why none of our patients have been cured by salvage treatments, although relapses was loco-regional only in 7. In part, this may also explain why pathologic prognostic criteria are unsatisfactory, the relapse being related to surgical inherent inadequacy much more than to the tumor inherent aggressiveness. On the other side, tumor relapse, whatever its cause, leads to the expression of pathologic markers of higher aggressiveness in all patients. As said above, this is all the more meaningful in our series, which selected late relapses.

Intriguingly, 3 out of 4 patients who were treated with adjuvant radiation therapy did not recur locally while experiencing late metastatic disease. The primary tumor arose from pleural site in 2 cases and from retroperitoneum in 1 case. The literature is inconclusive in regard of adjuvant RT in SFT [32-35]. In a series of 11 SFT treated with definitive RT without surgery, no patient had a local recurrence, and 9 were disease-free at 3 to 20 years from diagnosis [32]. Of course, RT can be hardly advocated in a tumor which, at least retrospectively, is “benign” in 70-80% of cases. However, prospective studies on adjuvant RT in SFT could be conceived when wide surgery is not feasible, as in meningeal, retroperitoneal and pleural presentations, and pathologic signs of “malignancy” are present at the onset.

Our series suggests that late relapses can occur in SFTs, even outside the meningeal setting. However, overall, they seem to be relatively rare. Thus, a prolonged follow-up may be advisable. More importantly, clinicians should be aware that new neoplastic lesions in a patient with a history of SFT can represent a malignant relapse with aggressive disease course, even though the primary tumor displayed “benign” features on pathologic assessment. Current treatment strategies of relapse are clearly insufficient, though reports of activity of new targeted therapies are now available [36-39] so that the outlook of the limited number of SFT patients who relapse may be due to improve in the next future.

## Abbreviations

SFT: Solitary fibrous tumor; MSFT: Malignant solitary fibrous tumor; FNAC: Fine needle aspiration cytology; pPNET: Peripheral primitive neuroectodermal tumor; FISH: Fluorescence in situ hybridation; RT: Radiation therapy; OS: Overall survival.

## Competing interest

The authors have no competing interests to declare.

## Authors’ contributions

SS and GGB conceived the study and the design. GGB and ML carried out data collection and AM performed statistical analysis. VM, PS and APDT performed pathologic review and immunoistochemical analysis. GA and PGC helped to draft the manuscript. All authors read and approved the final manuscript.
